# Supplementary material for: When Incorporated into Fruit Sorbet Matrix, Are the Fructans in Natural Raw Materials More Beneficial for Bone Health than Commercial Formulation Added Alone?
Source: Animals (Basel). 2022 Apr 28;12(9):1134. doi: 10.3390/ani12091134 (PMC9101039; doi:10.3390/ani12091134)
Supplement: Supplementary file 1 [file animals-12-01134-s001.zip › animals-1610405-supplementary.pdf]

Table S1. The content of growing rat diets (validation model and experimental groups).

Component (g/kg)	Groups of validation model		Experimental groups					
	RC	LC	LC-JA	LC-Y	LC-F	LC-JAS	LC-YS	LC-FS
corn starch	532.486	532.486	428.886	432.150	452.046	146.08	139.06	131.05
Cellulose	50.00	50.00	31.50	28.07	50.00	23.05	19.32	50.00
Sucrose	100.00	100.00	77.17	10.63	91.61	44.07	0.00	0.00
mineral mix	35.00	35.00	35.00	35.00	35.00	35.00	35.00	35.00
Jerusalem artichoke pulp	-	-	144.93	-	-	-	-	-
yacon root powder	-	-	-	211.64	-	-	-	-
Beneo Orafti Synergy 1	-	-	-	-	89.83	-	-	-
sorbet with Jerusalem artichoke pulp	-	-	-	-	-	470.30	-	-
sorbet with yacon root powder	-	-	-	-	-	-	524.11	-
sorbet with Beneo Orafti Synergy1	-	-	-	-	-	-	-	511.22

RC: diet with recommended calcium dose, LC: low-calcium diet (40% deficiency), LC-JA: low-calcium diet with Jerusalem artichoke pulp, LC-Y: low-calcium diet with yacon root powder, LC-F: low-calcium diet with Beneo Orafti Synergy 1, LC-JAS: low-calcium diet with sorbet containing Jerusalem artichoke pulp, LC-YS: low-calcium diet with sorbet containing yacon root powder, LC-FS: low-calcium diet with sorbet containing Beneo Orafti Synergy 1;

Content of casein (200 g/kg), soybean oil (70 g/kg), vitamin mix (10 g/kg), choline bitartate (2.5 g/kg), tert-butylhydroquinone (0.014 g/kg) was the same in all groups.

*Corn starch was placed in mineral mix instead of calcium
